# Fabrication and photoelectric conversion of densely packed C_60_–ethylenediamine adduct microparticle films-modified electrode covered with electrochemically deposited polythiophene thin-films†

**DOI:** 10.1039/d3ra05150a

**Published:** 2023-10-25

**Authors:** Shoto Banya, Yu Kumagawa, Daisuke Izumoto, Moyu Tanaka, Kengo Kanbe, Takeo Oku, Tsuyoshi Akiyama

**Affiliations:** a Division of Materials Science, Graduate School of Engineering, The University of Shiga Prefecture 2500, Hassaka Hikone Shiga 522-8533 Japan; b Division of Advanced Engineering Science, Graduate School of Engineering, The University of Shiga Prefecture 2500, Hassaka Hikone Shiga 522-8533 Japan; c Department of Materials Chemistry, School of Engineering, The University of Shiga Prefecture Hikone Shiga 522-8533 Japan akiyama.t@mat.usp.ac.jp

## Abstract

Polythiophene-modified densely packed C_60_–ethylenediamine adduct microparticle films were prepared using a combination of liquid–liquid interfacial precipitation of the adduct microparticles and electrochemical polymerization of 2,2′-bithiophene. The amount of polythiophene was varied as a function of scanning cycles of the applied potential during electrochemical polymerization. Fluorescence-emission properties of these composite films suggested the role of C_60_–ethylenediamine adduct microparticle film as a photosensitizer in addition to an electron acceptor for polythiophene. Furthermore, cathodic photocurrents were generated *via* excitation of C_60_–ethylenediamine adduct microparticle film and polythiophenes using the half-photocell properties of the electrode modified with composite film in the presence of methylviologen.

## Abstract 2

Electrodeposited polythiophene-modified C_60_–diamine adduct microparticle films generated photocurrent.

## Graphical abstract

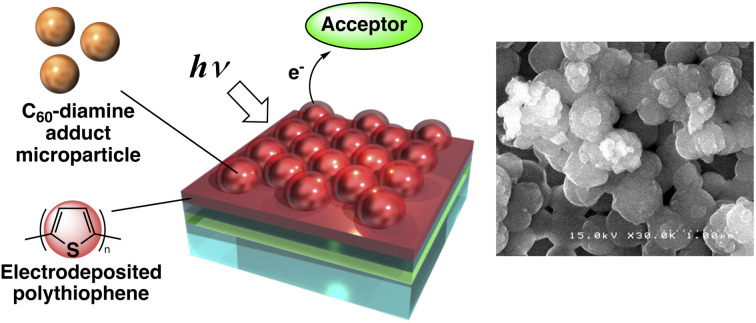

## Introduction

Fullerenes are well known sphere-shaped π-electron molecules,^1–3^ which have been receiving attention in the molecular based photoelectrochemical systems and organic-electronic devices owing to their electron acceptor and/or n-type semiconductor properties. In these applications, chemical modification of fullerenes is essentially important to obtain fullerene derivatives with adequate properties, such as redox potential, band structure, and solubility. One of the simplest methods used for the chemical modification of fullerenes is the addition reaction of alkylamines.^4–6^ Literature suggests the production of fullerene–diamine adducts *via* the addition reaction of C_60_ fullerene with a series of aliphatic diamines.^7,8^ Our research group has developed the fullerene–diamine adducts and evaluated their applications in photoelectric conversion.^9^ In preliminary studies, we reported the formation of sphere-shaped C_60_–aliphaticdiamine and rhombicdodecahedral-shaped C_70_–aliphaticdiamine adduct microparticles through simple mixing of individual solutions of fullerenes and diamines.^10,11^ The addition reaction of fullerenes and diamines is a key reaction to form these fullerene–diamine adduct microparticles, which has been suggested by elemental analysis, absorption spectra, infrared spectra, and X-ray diffraction patterns of corresponding adduct microparticles.^10–13^ These examples are interesting from a viewpoint of relationship to formation of unique anisotropic-shaped aggregates and/or assemblies of fullerenes or fullerene derivatives.^14–17^

C_60_–ethylenediamine adduct microparticles (C_60_P) can be prepared by simply mixing a toluene solution of C_60_ and another toluene solution of ethylenediamine at room temperature under usual atmosphere, which seems to be easy to scale up.^10,12,13,18^ C_60_P seems to consist of secondary microparticles composed of 2–3 nm-sized C_60_ and ethylenediamine adduct composite.^13^ C_60_P contains a residual amino group due to ethylenediamine.^12^ C_60_P function as electron acceptor for photoexciting donor molecules, such as polythiophene and porphyrin, owing to the photocurrent-generation and fluorescence-quenching properties of the composite films of C_60_P and polythiophene or porphyrin formed on electrodes.^18–20^ In addition, C_60_P or densely packed C_60_P film (C_60_PF) formed at liquid–liquid interface (liquid–liquid interfacial precipitation, LLIP),^19,21–24^ may work as photoexciting moiety because of the photocurrent-generation properties of the half-photocell containing C_60_PF on an electrode.^19^

Electrochemical polymerization is a widely used approach for the preparation of a conductive polymer thin-film on the electrode surface *via* electrolysis of an electrolyte-solution containing corresponding monomer molecule.^25^ The resultant conductive polymer thin-film is insoluble in the electrolyte solution. The amount of deposited polymer can be controlled by optimising the operating conditions of electrolysis during electrochemical polymerization. In addition, an electrochemically polymerized thin-film can be synthesized on electrodes of various shapes. Photovoltaic application is one of the common applications where electrochemically polymerised polythiophenes are used.^26–30^ Our research group investigated the preparation and photoelectric conversion of electrochemically polymerized polythiophene thin-films.^26,31–33^ Recently, a hierarchical double-layered polythiophene thin-films was synthesized using sequential electrochemical polymerization of corresponding 3,4-ethylenedioxythiophene and 2,2′-bithiophene monomers.^34–36^ In particular, the effect of the thickness of outer polybithiophene thin-film as photoexciting donor on the photoelectric conversion was investigated by controlling the electrochemical thickness of polybithiophene thin-films using their half-photocell properties.^35,36^ Such wet-type photoelectric conversion system is also interesting, as it is correlated with bio-inspired photoelectric conversion systems.^37–39^

Therefore, a systematic investigation of the photoelectrochemical properties of C_60_PF combined with polythiophene is crucial. Herein, a series of electrodeposited polybithiophene-modified C_60_PF/indium–tin-oxide (ITO) electrodes were fabricated and evaluated. The amount of polybithiophene was varied as a function of scanning cycles of applied potentials during electrochemical polymerization. The fluorescence-emission and photocurrent-generation properties of the modified electrodes were evaluated, and the role of C_60_P in C_60_PF was discussed.

## Experimental

**Fig. 1:**
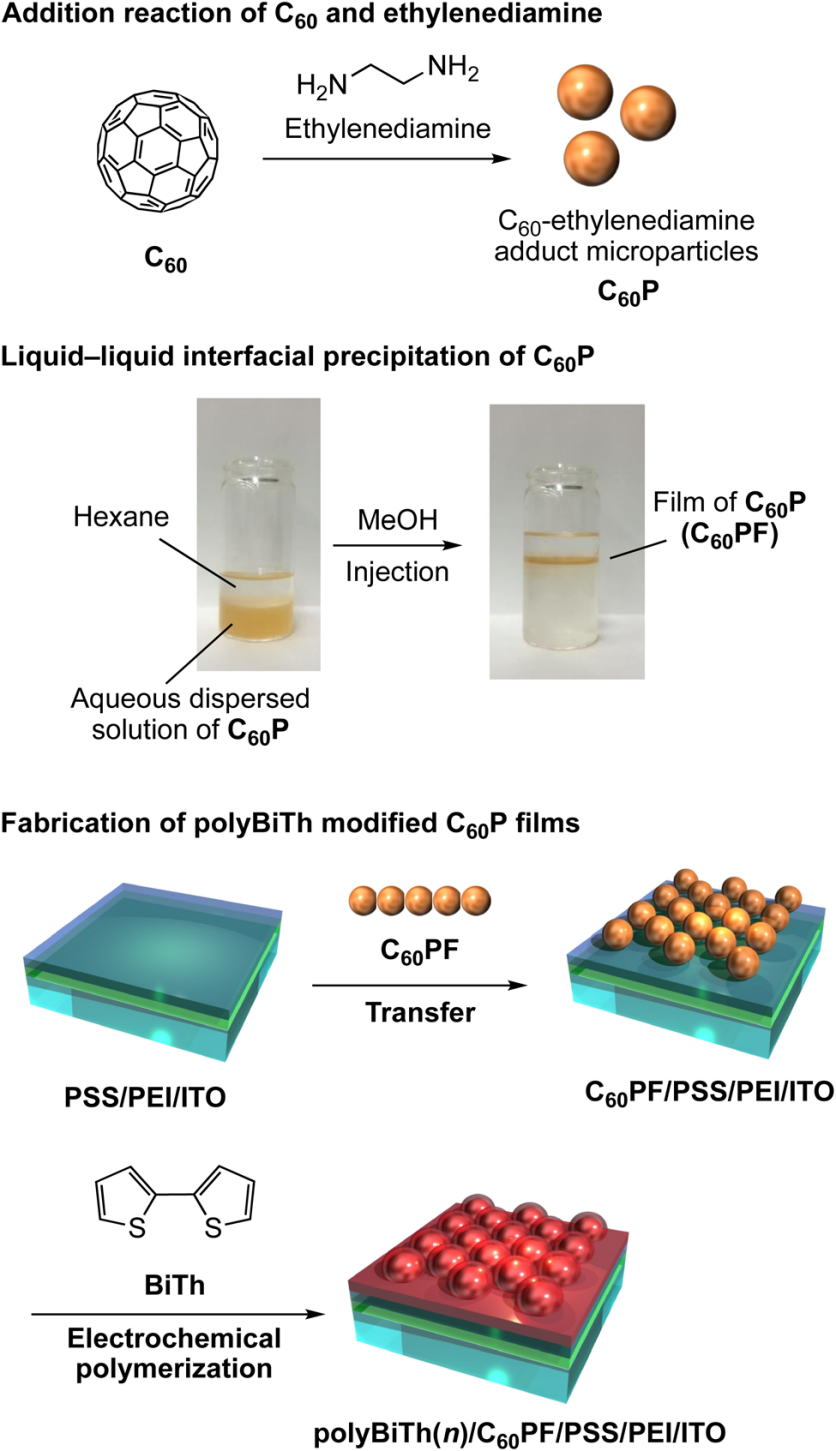
Fabrication of polybithiophene electrochemically polymerized thin-film modified densely packed C_60_–ethylenediamine adduct microparticles film.

All chemicals were commercially obtained and used as-received. Ultrapure water (18.2 M cm) was used in the experiments. A schematic of the fabrication procedure of polybithiophene-modified C_60_P film on an ITO electrode is shown in Fig. 1. The C_60_P and C_60_PF were synthesized as reported previously.^19^ To prepare C_60_P, individual solutions of C_60_ (2.0 mM, 144 mg/100 mL) and ethylenediamine (2.0 M, 13.4 mL/100 mL) were prepared in toluene and mixed for 1 min under sonication at room temperature, followed by continuous stirring for 20 h at room temperature. The resultant submicrometer-sized particles of C_60_P were collected after centrifugation (6000 rpm, 30 min) and dried in vacuum to obtain C_60_P as a brown powder. An ITO-coated glass substrate (ITO, Geomatec 0002, with a sheet resistance of *ca.* 10 Ω per square) was used as a transparent electrode. The substrate was washed using acetone and methanol in each sequence for 10 min under ultrasonic treatment and dried using nitrogen gas. Subsequently, the ITO electrode was cleaned in an ozone atmosphere (using UV ozone cleaner) for 15 min. The pre-cleaned ITO substrate was immersed in an aqueous solution of 1.5 mM polyethyleneimine (PEI, MW: 750 000) for 30 min at room temperature, followed by washing with ultrapure water to modify the PEI on the ITO surface, denoted as PEI/ITO. The PEI/ITO substrate was immersed in an aqueous solution of 0.5 mM poly(styrene sulfonate) (PSS, MW: 70 000) for 30 min at room temperature, followed by washing with ultrapure water to modify PSS on the PEI/ITO surface, denoted as PSS/PEI/ITO.^40,41^ The PSS/PEI/ITO was stored in ultrapure water at room temperature for subsequent use.

The C_60_P film is prepared by LLIP method as follows: a liquid–liquid interface using a 0.2 mg mL^−1^ aqueous dispersed solution of C_60_P (15 mL) and hexane (10 mL) was prepared in a vial (*ϕ* = 30 mm, volume: 50 mL). Immediately after, methanol (15 mL) as aggregation inducer of C_60_P, was injected into the solution vigorously, resulting in an instantaneous change in the color of the aqueous phase from brown to colorless. Simultaneously, a liquid-like film of C_60_Ps forms at the hexane–water interface. The resultant C_60_PF was manually transferred onto the surface of the PSS/PEI/ITO by vertical lifting to obtain C_60_PF on ITO electrode denoted as C_60_PF/PSS/PEI/ITO or polyBiTh(*n*)/C_60_PF/PSS/PEI/ITO (*n* = 0).

The C_60_PF/PSS/PEI/ITO, a nickel plate, and silver wire were used as a working, counter, and reference electrodes, respectively, in a three-electrode electrochemical cell containing 1.0 mM 2,2′-bithiophene (BiTh), and 0.1 M *n*Bu_4_NPF_6_ in CH_2_Cl_2_. The distance between working and counter electrodes is fixed at 5 mm. Immersed dimension of the working electrode in an electrolyte solution is 2 cm × 1.5–1.8 cm. The applied potential to the working electrode was scanned between 0 and +2 V (initial potential 0 V, switching potential +2 V and final potential 0 V) *vs.* silver wire for *n* times at a speed of 0.05 V s^−1^ using the cyclic voltammetry (CV) method of a potentiostat (ALS, model 650C), where *n* is the number of sweeping potentials. During electrochemical polymerization, the electrolyte solution was stirred at a speed of 400 rpm. After electrochemical polymerization, the working electrode was removed from the electrolyte solution followed by rinsing with enough amount of CH_2_Cl_2_ and drying in air to obtain the polybithiophene (polyBiTh)-modified C_60_PF on ITO electrode, denoted as polyBiTh(*n*)/C_60_PF/PSS/PEI/ITO (*n* = 1, 3, 5, 13, 20, and 40 cycles). The polyBiTh-modified PSS/PEI/ITO electrodes without C_60_PF were also prepared using same polymerization condition of BiTh, white are denoted as polyBiTh(*n*)/PSS/PEI/ITO (*n* = 1, 3, 5, 13, 20, and 40 cycles).

Transparent Ultraviolet-Visible-Near Infrared (UV-vis-NIR) spectra of the polyBiTh(*n*)/C_60_PF/PSS/PEI/ITO were recorded on Jasco V-670 UV-vis-NIR spectrometer to investigate their optical properties. Fluorescence spectra of the polyBiTh(*n*)/C_60_PF/PSS/PEI/ITO were measured using Jasco FP-6600. Excitation light was irradiated on polyBiTh side at an incident angle of 45° and fluorescence emission was measured at an exit angle of 45°. Scanning electron micrographs (SEM) for the polyBiTh(*n*)/C_60_PF/PSS/PEI/ITO were recorded using a Hitachi S-4500 scanning electron microscope. Photocurrent measurements were carried out in a three-electrode photoelectrochemical cell using a potentiostat (Huso, HECS 318C) and a monochromatic light from a 150 W xenon lamp (Bunko Keiki, SM-25). The polyBiTh(*n*)/C_60_PF/PSS/PEI/ITO (*n* = 0–40 cycles), an Ag/AgCl (sat. KCl), and a platinum wire were used as working, reference, and counter electrodes, respectively. The aqueous solution containing 0.1 M sodium perchlorate and 1.0 mM 1,1′-dimethyl-4,4′-bipyridinium dichloride hydrate (methylviologen, MV^2+^) as electron acceptors was employed as an electrolyte. Photocurrents were measured at an applied potential of *E* = 0 or −0.2 V *vs.* Ag/AgCl (sat. KCl) for the working electrode. The monochromatic light was irradiated to the working electrode. The irradiated area of the working electrode was adjusted to be 0.38 cm^2^. The incident photon-to-current conversion efficiency (IPCE) of the photocurrent was evaluated at the given absorbance using the following eqn (1).1
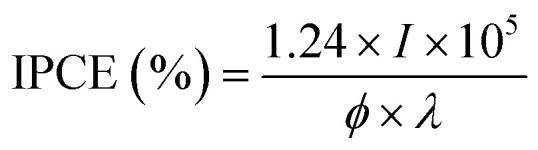
where *λ* (nm) is irradiation wavelength, *I* (A cm^−2^) is photocurrent density, *ϕ* (W cm^−2^) is irradiation light power. Applied potential dependence of photocurrents under irradiation of white light from a 150 W xenon lamp with ultraviolet cut-off filter (Bunko Keiki, SM-25).

## Results and discussion

**Fig. 2:**
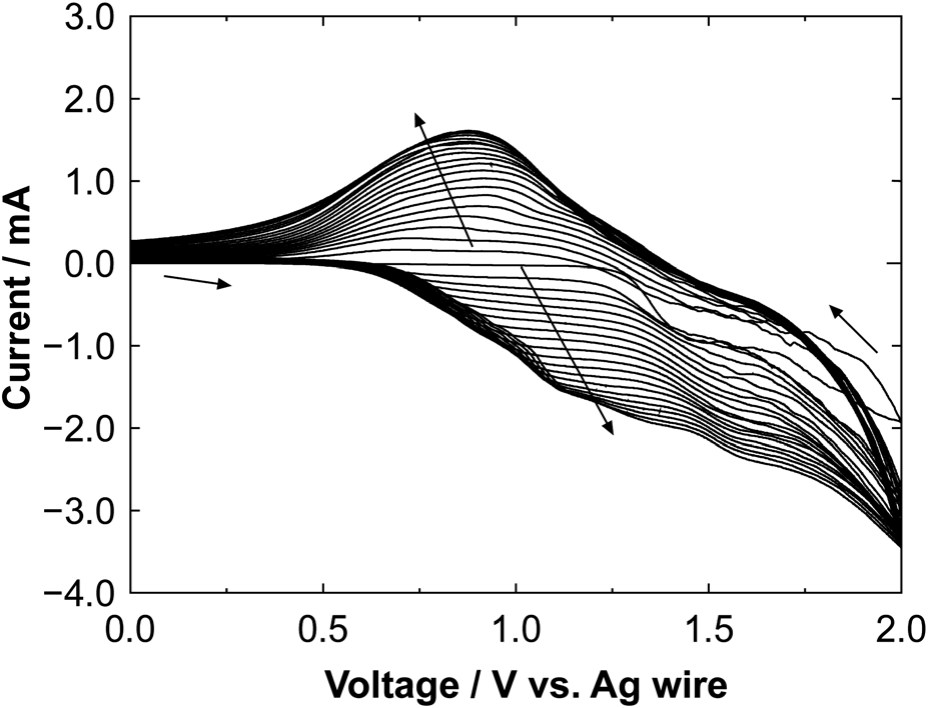
Cyclic voltammogram of the electrochemical polymerization of polyBiTh on C_60_PF/PSS/PEI/ITO during preparation of polyBiTh(20)/C_60_PF/PSS/PEI/ITO.

Fig. 2 shows a cyclic voltammogram obtained during the electrochemical polymerization of polyBiTh on C_60_PF/PSS/PEI/ITO to prepare polyBiTh(20)/C_60_PF/PSS/PEI/ITO. The values of oxidation and reduction current increase with increasing number of scanning cycles (*n*) of applied potential, indicating the formation of polyBiTh *via* electrochemical oxidation of BiTh on C_60_PF/PSS/PEI/ITO followed by re-reduction. Furthermore, essentially similar behavior is also observed in all polyBiTh(*n*)/C_60_PF/PSS/PEI/ITO (*n* = 1–40 cycles), as shown in Fig. S1.† These voltammetric characteristics of the polyBiTh(*n*)/C_60_PF/PSS/PEI/ITO are essentially similar to that of polyBiTh film prepared using electrochemical polymerization of BiTh on a bare electrode (Fig. S2†). Moreover, no obvious changes in the current profile of C_60_PF/PSS/PEI/ITO (Fig. S3†) were observed in the cyclic voltammogram when a potential sweep is applied in the 0.1 M *n*Bu_4_NPF_6_ in CH_2_Cl_2_ without BiTh, indicating the stability of C_60_PF during electrochemical preparation of polyBiTh.

**Fig. 3:**
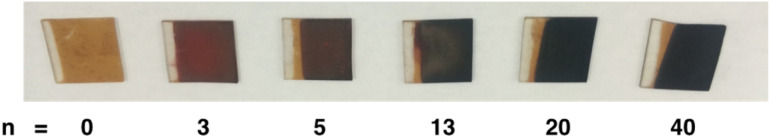
Photographs of polyBiTh(*n*)/C_60_PF/PSS/PEI/ITO (*n* = 0, 3, 5, 13, 20, and 40 cycles).

Photographs of polyBiTh(*n*)/C_60_PF/PSS/PEI/ITO (*n* = 0, 3, 5, 13, 20, and 40 cycles) is shown in Fig. 3. A light brown colored layer is observed in the C_60_PF/PSS/PEI/ITO. After electrochemical polymerization, the color of the adsorbed films changes slightly. In addition, the color of the films becomes darker with increasing *n* at the given applied potential, suggesting an increase in the adsorbed amount of polyBiTh with increasing *n*. In the case of polyBiTh(*n*)/PSS/PEI/ITO without C_60_PF, similar change tendency of the color of the films is observed (Fig. S4†).

**Fig. 4:**
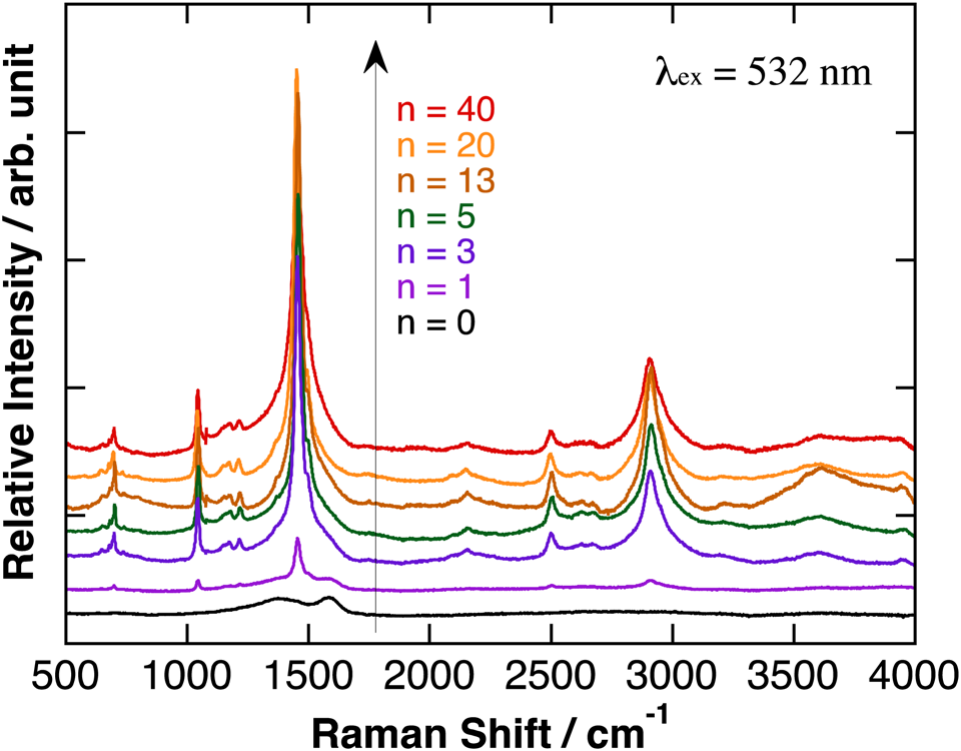
Relative Raman scattering spectra of polyBiTh(*n*)/C_60_PF/PSS/PEI/ITO, after baseline correction and adjusted for comparison. Relative intensities of Raman signals were not adjusted.

Relative Raman scattering spectra of polyBiTh(*n*)/C_60_PF/PSS/PEI/ITO are shown in Fig. 4. The spectra were obtained after baseline correction of Raman scattering spectra as measured (Fig. S5†). In Fig. 4, positions of each baseline were adjusted for comparison, and relative intensities of Raman signals were not adjusted. The characteristic Raman scattering signals are observed at a Raman shift of 1045, 1455, 2500, and 2905 cm^−1^ after the electrochemical modification of polyBiTh rather than the broad Raman scattering signal of C_60_PF/PSS/PEI/ITO at 1410 and 1590 cm^−1^. These results suggest the formation of polyBiTh on C_60_PF/PSS/PEI/ITO *via* electrochemical polymerization.

**Fig. 5:**
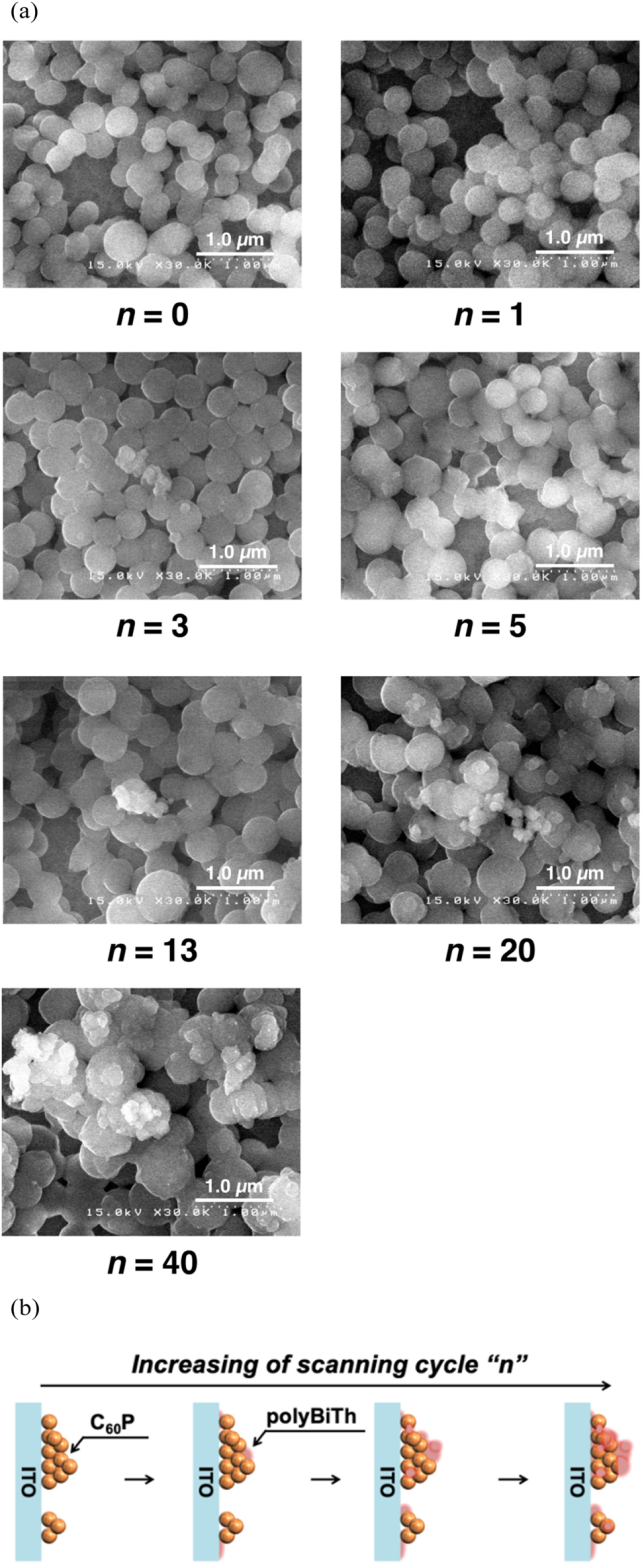
(a) SEM images of polyBiTh(*n*)/C_60_PF/PSS/PEI/ITO (*n* = 0, 1, 3, 5, 13, 20, and 40 cycles), and (b) proposed mechanism of polyBiTh thin-films growing on C_60_PF/PSS/PEI/ITO.

Fig. 5(a) and S6(a)† show the SEM images of polyBiTh(*n*)/C_60_PF/PSS/PEI/ITO (*n* = 1, 3, 5, 13, 20, and 40 cycles). Sphere-shaped structures with diameters of 350–500 nm are observed, which can be attributed to C_60_P. An approximate evaluation of the diameters of C_60_P present in the polyBiTh(*n*)/C_60_PF/PSS/PEI/ITO indicates an increase in the diameters of C_60_P with increasing *n*, thereby suggesting the adsorption of polyBiTh on the entire surface of C_60_P present in C_60_PF/PSS/PEI/ITO. In addition, structures of a few tens to 100 nm size are observed at all values of *n* except *n* = 1. The growth of these microstructures is non-uniform on polyBiTh, which can be attributed to the considerably small protuberance of the concentration of electric field on C_60_P or polyBiTh and resultant acceleration of electrochemical polymerization of BiTh at the site. This is supported by the cross-sectional SEM images of polyBiTh(*n*)/C_60_PF/PSS/PEI/ITO, which are shown in Fig. S6(b).†

Thus, the suggested mechanism can be a combination of the following: (1) uniform formation of polyBiTh thin-film on the C_60_P surface, and (2) non-uniform formation of polyBiTh structures of a few tens to 100 nm size, which is shown in Fig. 5(b).

**Fig. 6:**
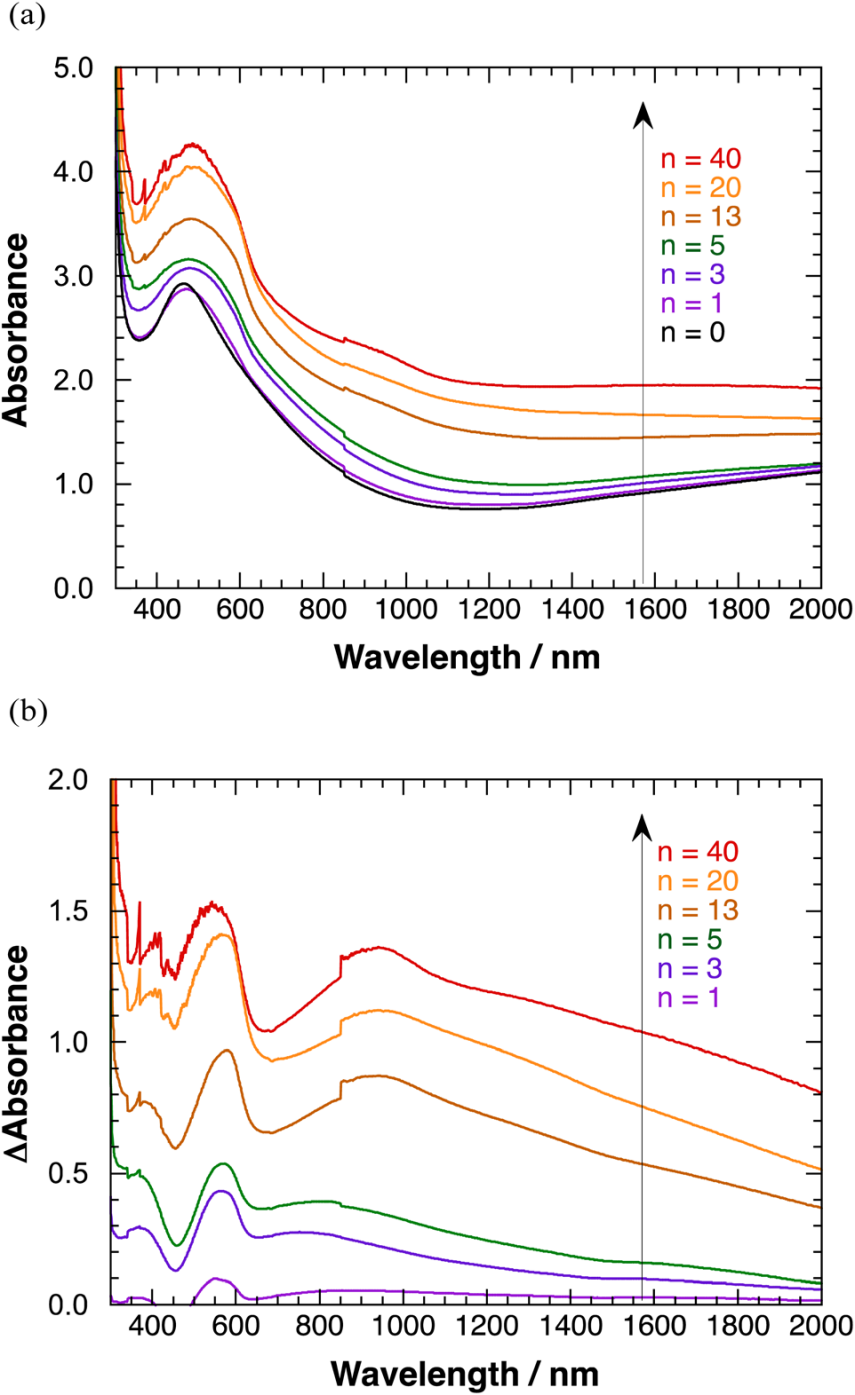
(a) Transmission absorption spectra of polyBiTh(*n*)/C_60_PF/PSS/PEI/ITO, and (b) differential transmission absorption spectra of polyBiTh(*n*)/C_60_PF/PSS/PEI/ITO after subtraction of C_60_PF/PSS/PEI/ITO absorption spectra.

Transparent absorption spectra of polyBiTh(*n*)/C_60_PF/PSS/PEI/ITO are shown in Fig. 6(a). Discontinuous steps in the absorption spectra were observed at 850 nm, which correspond to the switching wavelength during spectral measurements. These obvious spectral steps may be due to the significant effect of 350 nm-sized C_60_Ps in C_60_PF on the light path. A broad absorption band at a peak wavelength of 460 nm is observed when *n* = 0 (C_60_PF/PSS/PEI/ITO), which is attributed to C_60_P present in C_60_PF.^18,19^ Furthermore, the absorbance values increase for the broad absorption band at a peak wavelength of approximately 500 nm and wavelength region longer than 800 nm with increasing value of *n* in the range of 1–40. An increase in the baseline, which is the sum of optical absorption and geometrical scattering, with increasing *n* is reasonable owing to the combination of C_60_PF and polyBiTh. Differential absorption spectra of polyBiTh(*n*)/C_60_PF/PSS/PEI/ITO (*n* = 1–40 cycles) obtained after subtraction of the absorption spectrum of C_60_PF/PSS/PEI/ITO from corresponding absorption spectra of polyBiTh(*n*)/C_60_PF/PSS/PEI/ITO are shown in Fig. 6(b). A broad absorption band at 450–650 nm is observed in all polyBiTh-modified electrode because of the neutral state of typical polythiophene. The absorbance values increase with increasing *n*, suggesting an increase in the adsorbed amount of polyBiTh on C_60_PF/PSS/PEI/ITO with increasing *n*. In addition, a significantly broad NIR absorption band at a peak wavelength of approximately 850–950 nm is observed in all polyBiTh-modified electrode, which is attributed to the polaron absorption band of polythiophenes. A relatively higher absorbance of the polaron absorption band than the visible neutral absorption band of polyBiTh is observed more significantly at *n* = 13, 20, and 40 cycles than those at *n* = 1, 3, and 5 cycles. This is attributed to the residual polaron of polyBiTh during electrochemical polymerization process. Although the applied potential is set to 0 V *vs.* Ag wire at the end of electrochemical polymerization, some amount of polyBiTh cannot be reduced electrochemically. A relatively higher ratio of oxidized state of polyBiTh is observed in the polyBiTh film when a relatively larger amount of polyBiTh is formed at *n* = 13, 20, and 40 cycles than that at *n* = 1, 3, and 5 cycles. Corresponding absorption spectra of polyBiTh(*n*)/PSS/PEI/ITO without C_60_PF are shown in Fig. S7(a) and (b).† Characteristic absorption bands of the neutral state and polaron of the typical polythiophene are also observed. Differences of absorbance in differential absorption spectra of polyBiTh(*n*)/C_60_PF/PSS/PEI/ITO and polyBiTh(*n*)/PSS/PEI/ITO may be due to difference of surface morphologies at polyBiTh film forming.

**Fig. 7:**
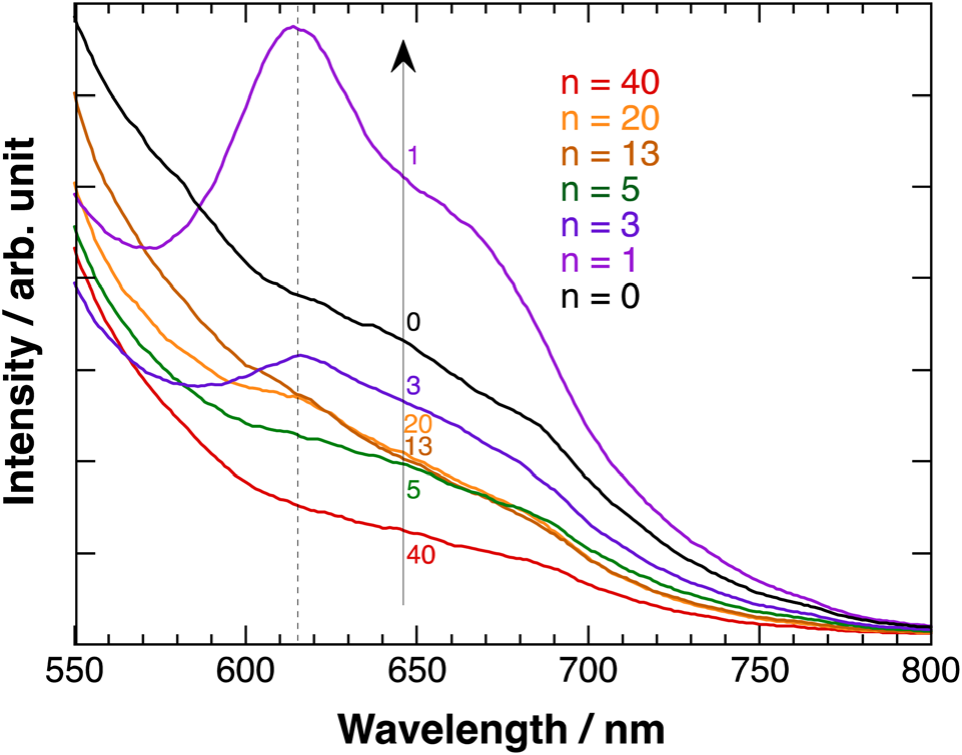
Fluorescence emission spectra of polyBiTh(*n*)/C_60_PF/PSS/PEI/ITO, *λ*_ex_ = 450 nm.

Fig. 7 shows the fluorescence emission spectra of polyBiTh(*n*)/C_60_PF/PSS/PEI/ITO exited at 450 nm. A considerably broad emission in the wavelength region of 600–700 nm with inflection points at 640 and 685 nm is observed at *n* = 0 (C_60_PF/PSS/PEI/ITO). In contrast, a decrease in the emission with increasing wavelength is observed at all other values of *n*. The broad emission band at 600–700 nm is attributed to the fluorescence-emission from the excited state of C_60_P present in the C_60_PF/PSS/PEI/ITO. Furthermore, two characteristic emission peaks at approximately 615 and 670 nm are observed in polyBiTh(1)/C_60_PF/PSS/PEI/ITO, which are attributed to the photoexcited state of neutral polythiophene.^20,42^ Order of emission intensity at 615 nm of polyBiTh(*n*)/C_60_PF/PSS/PEI/ITO (*n* = 1–40 cycles) is given as follows:*n* = 1 > 3 > 13, 20 > 5 > 40 cycles

This order seems to be a sum of some mechanisms of increasing or decreasing of emission from excited polyBiTh, shown as follows. It is known that polaron decreases emission from excited state of polythiophene during the fluorescence-emission of polythiophene.^43^ The absorption spectra recorded in the present study indicates a higher effect of polaron on the polyBiTh film with a larger *n* than that on polyBiTh film with a smaller *n*. Another reason for the decreased emission can be diffusion and multiple reflections of light owing to the changes in surface morphology of polyBiTh(*n*)/C_60_PF/PSS/PEI/ITO. Furthermore, emission of polyBiTh can be enhanced by the energy transfer from photoexcited C_60_PF to polyBiTh. In addition, if the C_60_P has a refractive index similar to C_60_, the irradiated light may be localised in the area of several tens of nm in the proximity of C_60_P, thereby enhancing the excitation of C_60_P and polyBiTh. Thicker polyBiTh film could be formed at higher values of *n*. The degree of emission of polyBiTh induced by energy transfer from C_60_PF decreases with the increasing *n*. Furthermore, the degree of electron-transfer quenching, which can happen simultaneously, from the excited state of polyBiTh to C_60_PF,^20^ decreases with the increasing *n*. Thus, a combination of all the aforementioned mechanisms explains the order of the emission intensity of polyBiTh(*n*)/C_60_PF/PSS/PEI/ITO.

**Fig. 8:**
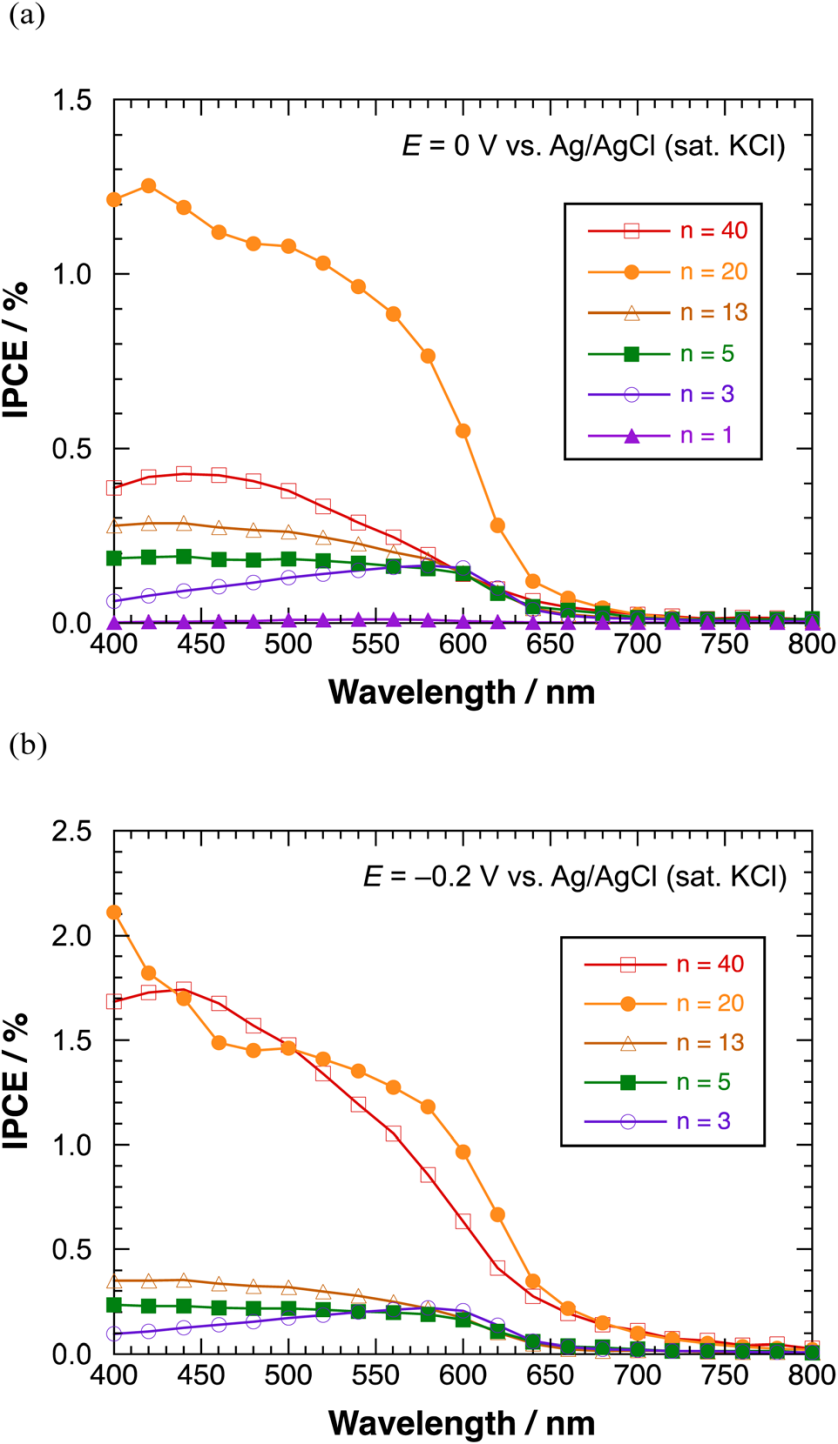
IPCE profiles of polyBiTh(*n*)/C_60_PF/PSS/PEI/ITO in the presence of methyl viologen at (a) 0 V, and (b) −0.2 V *versus* Ag/AgCl (sat. KCl).

Fig. 8(a) shows the IPCE profiles for the cathodic photocurrent of polyBiTh(*n*)/C_60_PF/PSS/PEI/ITO (*n* = 1–40 cycles) at *E* = 0 V. The IPCEs of polyBiTh(*n*)/C_60_PF/PSS/PEI/ITO (*n* = 1, 3, 5, 13, and 20 cycles) increase with the increasing *n* because of the increasing contribution of the increased amount of polyBiTh. Furthermore, the IPCEs increased in the wavelength region of 400–550 nm at *n* = 5, 13, and 20 compared to that of polyBiTh(3)/C_60_PF/PSS/PEI/ITO, which is attributed to the increasing amount of polyBiTh at a distance from C_60_P of C_60_PF/PSS/PEI/ITO, and the increasing amount of excited polyBiTh that contributes in the generation of cathodic photocurrent. These IPCE profiles change can be explained as the result of a combination of the following photocurrent generation mechanisms and electron transfer reactions: (1) cathodic photocurrent generation due to the electron-transfer from excited polyBiTh to MV^2+^ in bulk, (2) reduction of cathodic photocurrent due to electron-transfer from excited polyBiTh to C_60_P of C_60_PF, and (3) an enhanced generation of excited polyBiTh *via* energy transfer from photoexcited C_60_P and/or light concentration around C_60_P (as described above). In particular, the enhanced generation of excited polyBiTh is induced by absorption of C_60_P of C_60_PF/PSS/PEI/ITO, thereby reducing the cathodic photocurrent in the wavelength region of 400–550 nm.

Moreover, the IPCEs of polyBiTh(40)/C_60_PF/PSS/PEI/ITO are considerably lower than that of polyBiTh(20)/C_60_PF/PSS/PEI/ITO due to the suppressed electron-transfer by thicker polyBiTh film of polyBiTh(40)/C_60_PF/PSS/PEI/ITO. Fig. 8(b) shows the IPCE profiles of polyBiTh(*n*)/C_60_PF/PSS/PEI/ITO (*n* = 3, 5, 13, 20, and 40 cycles) at *E* = −0.2 V. The IPCE values of these modified electrodes are higher than those at *E* = 0 V. Thus, the overall IPCE profile (except their values) for irradiation wavelength does not change at *E* = 0 V. However, IPCEs of polyBiTh(40)/C_60_PF/PSS/PEI/ITO increase significantly and become similar to that of polyBiTh(20)/C_60_PF/PSS/PEI/ITO. This is attributed to the decrease in polaron of polyBiTh of polyBiTh(40)/C_60_PF/PSS/PEI/ITO owing to more negative applied potential −0.2 V than 0 V. This results in increasing neutral moiety of polyBiTh, which may increase the cathodic photocurrent.

**Fig. 9:**
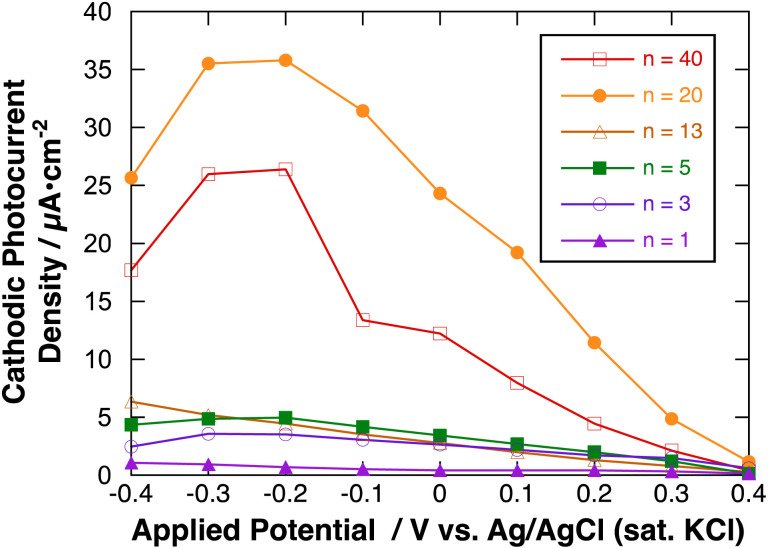
Applied potential dependence of cathodic photocurrent of polyBiTh(*n*)/C_60_PF/PSS/PEI/ITO.

**Fig. 10:**
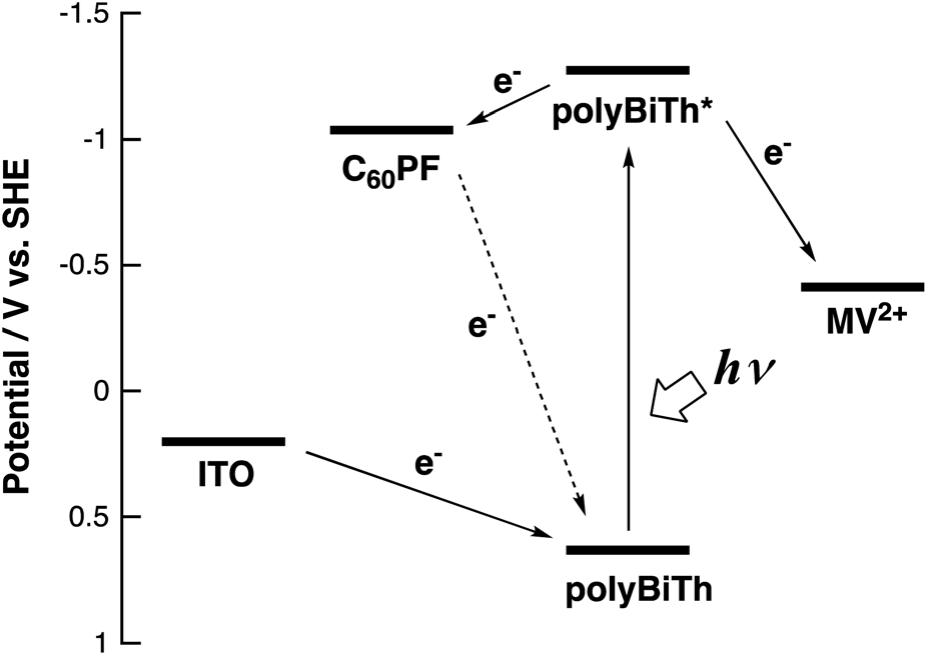
Photocurrent energy diagram.

Fig. 9 shows the effect of applied potential on the photocurrent of polyBiTh(*n*)/C_60_PF/PSS/PEI/ITO (*n* = 1–40) under irradiation of white light from a 150 W xenon lamp with ultraviolet cut-off filter. Cathodic photocurrent increases with increasing values of the negative applied potentials in the region from 0.4 to −0.2 V. The cathodic photocurrent decreases in the region of −0.3 to −0.4 V with higher negative potentials. This may be affected by the static cathodic current of electrochemical reduction of MV^2+^, which cannot be ignored. The aforementioned photocurrent-generation properties suggest that the photocurrent-generation mechanism of polyBiTh(*n*)/C_60_PF/PSS/PEI/ITO is a combination of sequential reactions of electron-transfer and energy-transfers (Fig. 10).^19,36^

These cathodic photocurrents are generated by electron-transfer through C_60_P of a size of submicrometers. In general, long-range electron-transfer is difficult to take place. In fact, C_60_P consists of polymer or oligomer of C_60_–ethylenediamine adduct. The C_60_ moieties of C_60_P are considerably close to each other, thereby facilitating a smooth electron-transfer among C_60_Ps within C_60_P.

This study suggests the unique photochemical and electrochemical properties of C_60_P present in the C_60_PF/PSS/PEI/ITO based on the evaluation of their effect on polyBiTh moiety for the fluorescence and photocurrent-generation properties of the modified electrodes. A detailed photochemical analysis, including the time-resolved photochemical spectrum measurements and photoelectrochemical properties, of C_60_P and their derivatives is required to further illustrate the role of C_60_P under photoexcitation. This will be a part of our ongoing research work.

## Conclusions

Composite films consisting of electrochemically polymerized polythiophene and densely packed C_60_–ethylenediamine adduct microparticle film were synthesized on electrodes. Fluorescence-emission and photocurrent-generation properties of these modified electrodes indicated the role of C_60_–ethylenediamine adduct microparticle film as photoexciting moiety in addition to an electron acceptor for polythiophene which could transfer excitation energy to polythiophene. These unique properties of C_60_–ethylenediamine adduct microparticle film suggested the potential applications of the synthesized film in photoelectrochemical devices, such as photoelectric conversion devices and sensors.

## Conflicts of interest

There are no conflicts to declare.

## Supplementary Material

RA-013-D3RA05150A-s001
