# Supplementary material for: Fabrication and photoelectric conversion of densely packed C60–ethylenediamine adduct microparticle films-modified electrode covered with electrochemically deposited polythiophene thin-films
Source: RSC Adv. 2023 Oct 25;13(44):31244–51. doi: 10.1039/d3ra05150a (PMC10599220; doi:10.1039/d3ra05150a)
Supplement: RA-013-D3RA05150A-s001 [file RA-013-D3RA05150A-s001.pdf]

Electronic Supplementary Material (ESI) for RSC Advances.
This journal is © The Royal Society of Chemistry 2023

Electronic supplementary information

Fabrication and photoelectric conversion of densely packed C_{60}-ethylenediamine adduct microparticle films-modified electrode covered with electrochemically deposited polythiophene thin-films

Shoto Banya,[a], Yu Kumagawa,[a] Daisuke Izumoto,[a] Moyu Tanaka,[a] Kengo Kanbe,[b] Takeo Oku[c], and Tsuyoshi Akiyama[c]*

[a] *Division of Materials Science, Graduate School of Engineering, The University of Shiga Prefecture, 2500, Hassaka, Hikone, Shiga 522-8533, Japan*

[b] *Division of Advanced Engineering Science, Graduate School of Engineering, The University of Shiga Prefecture, 2500, Hassaka, Hikone, Shiga 522-8533, Japan*

[c] *Department of Materials Chemistry, School of Engineering, The University of Shiga Prefecture, Hikone, Shiga 522-8533, Japan*

E-mail: akiyama.t@mat.usp.ac.jp (Tsuyoshi Akiyama)

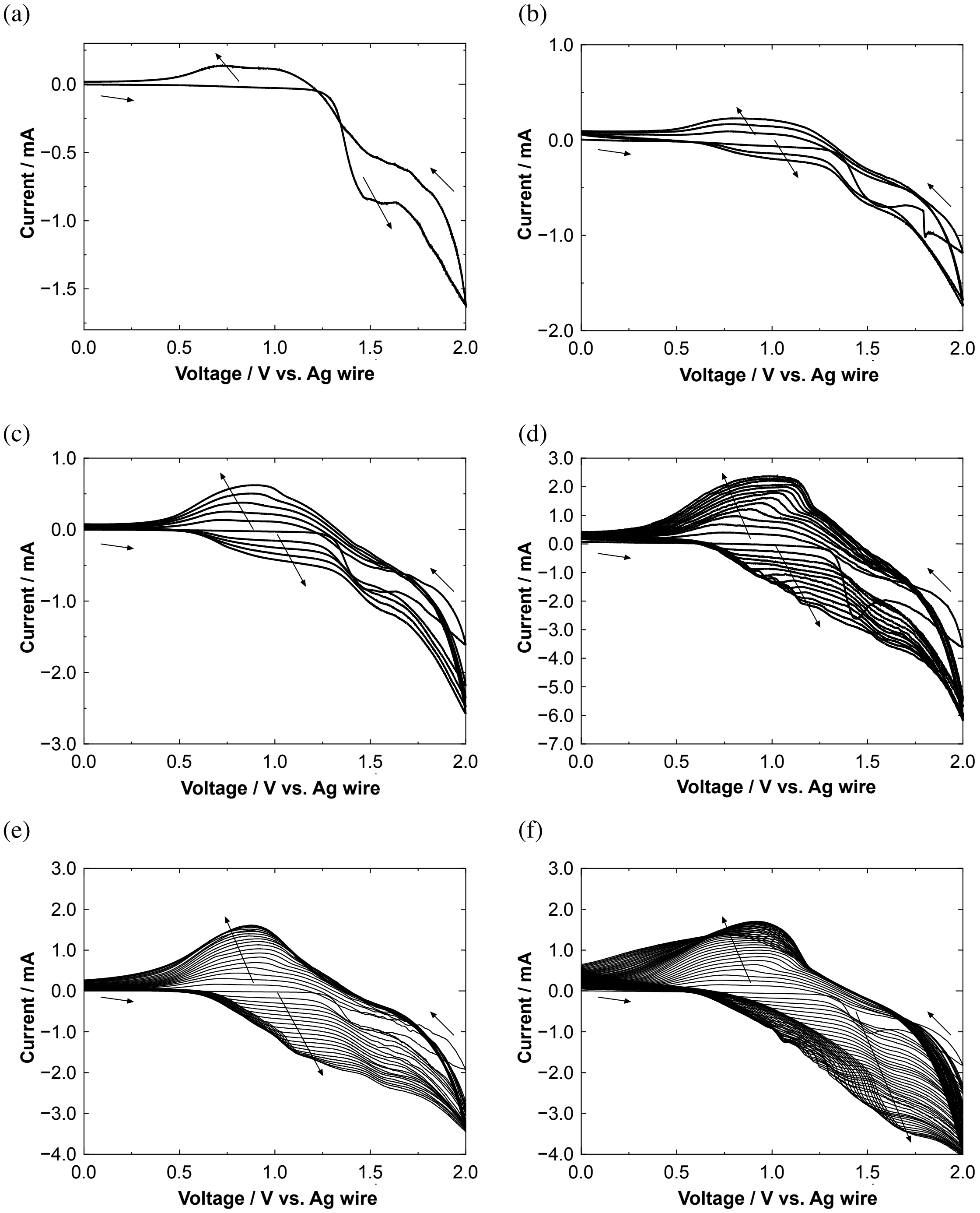

Fig. S1. Cyclic voltammograms of polyBiTh(*n*)/C_{60}PF/PSS/PEI/ITO in electrochemical polymerization of 2,2’-bithiophene; (a) ~ (f) for *n* = 1, 3, 5, 13, 20 and 40, respectively. Voltammogram (e) is also shown in Fig. 2.

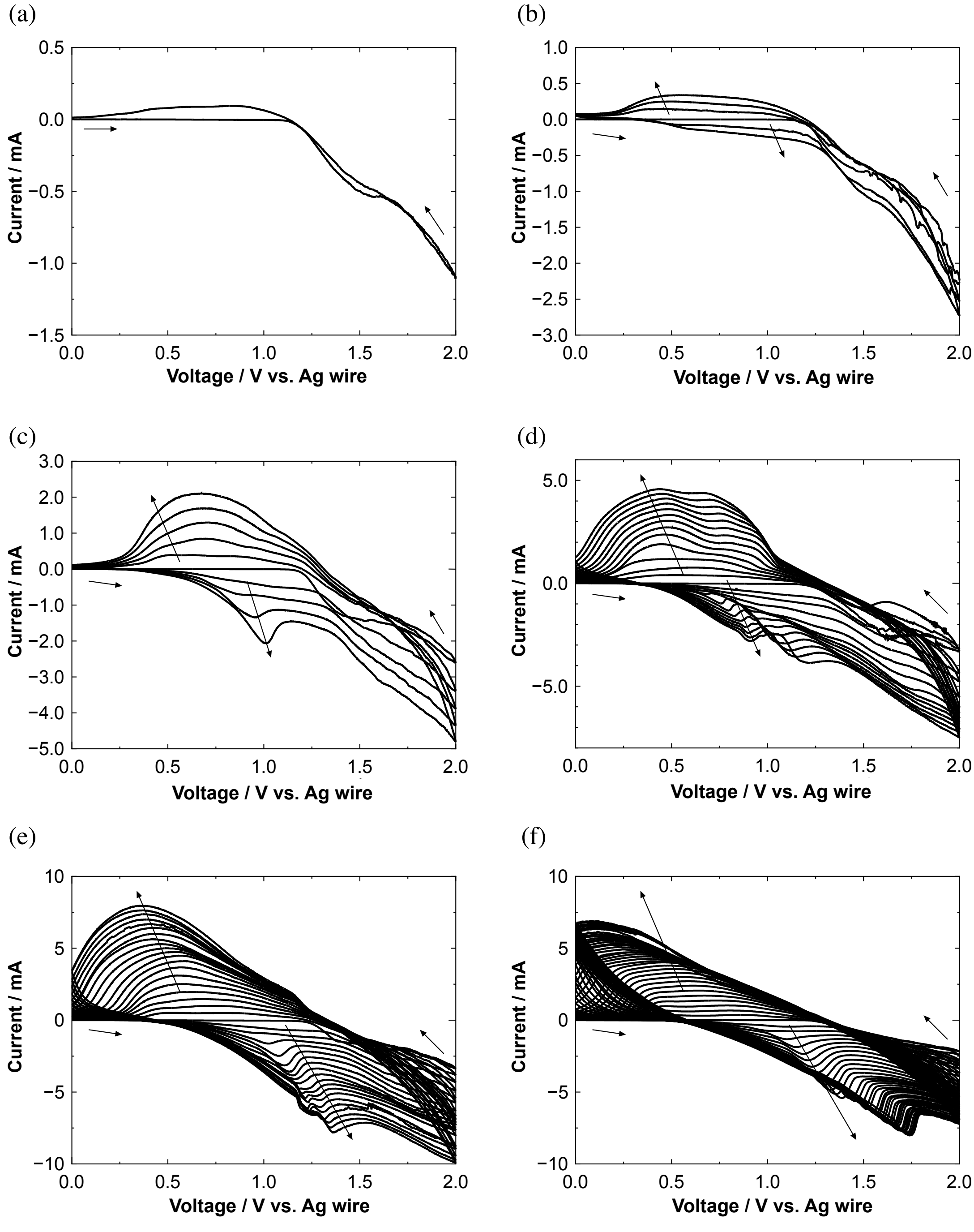

Fig. S2. Cyclic voltammograms of polyBiTh(*n*)/PSS/PEI/ITO in electrochemical polymerization of 2,2'-bithiophene; (a) ~ (f) for *n* = 1, 3, 5, 13, 20 and 40, respectively.

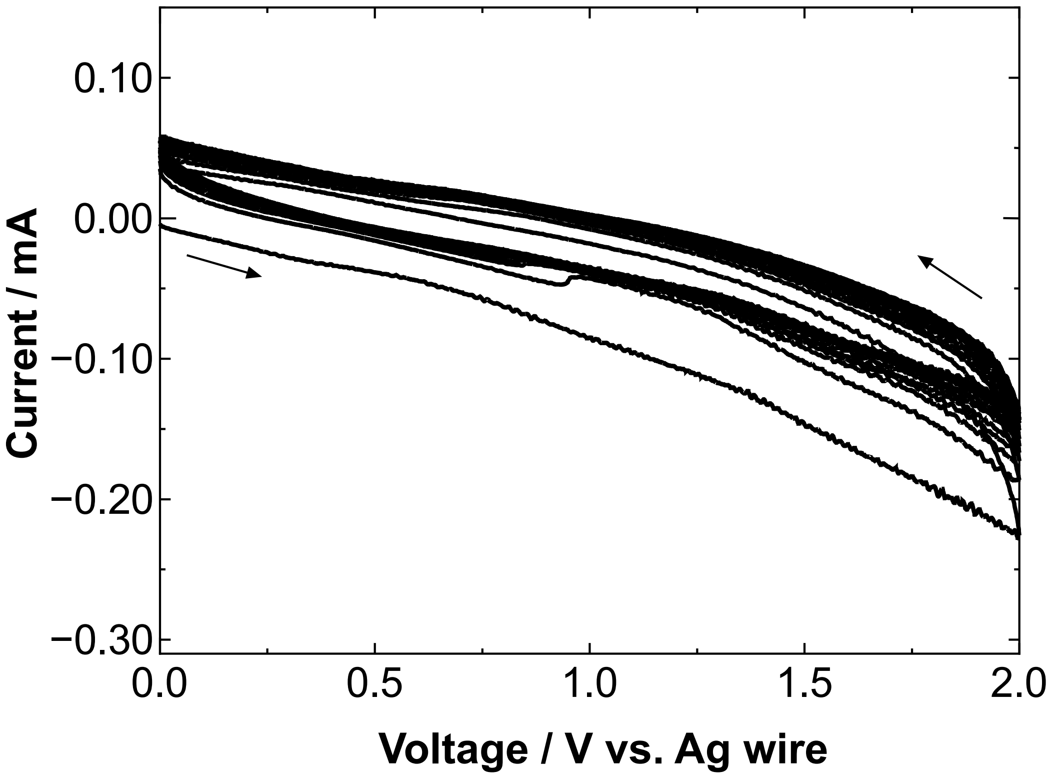

Fig. S3. Cyclic voltammograms of C_{60}PF/PSS/PEI/ITO. Cyclic voltammogram were measured in 1,2–dichloroethane containing 0.1 M *n*-Bu_4NPF_6 as supporting electrolyte at room temperature in 20 cycles.

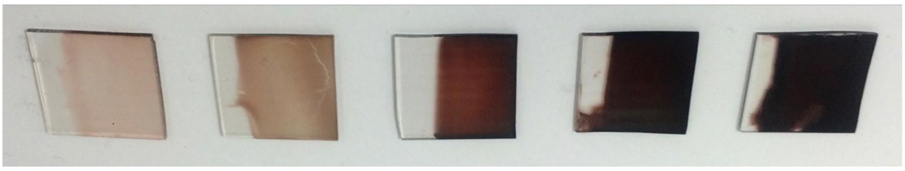

n = 1 3 5 13 20

Fig. S4. Photographs of polyBiTh(*n*)/PSS/PEI/ITO (*n* = 1, 3, 5, 13, and 20).

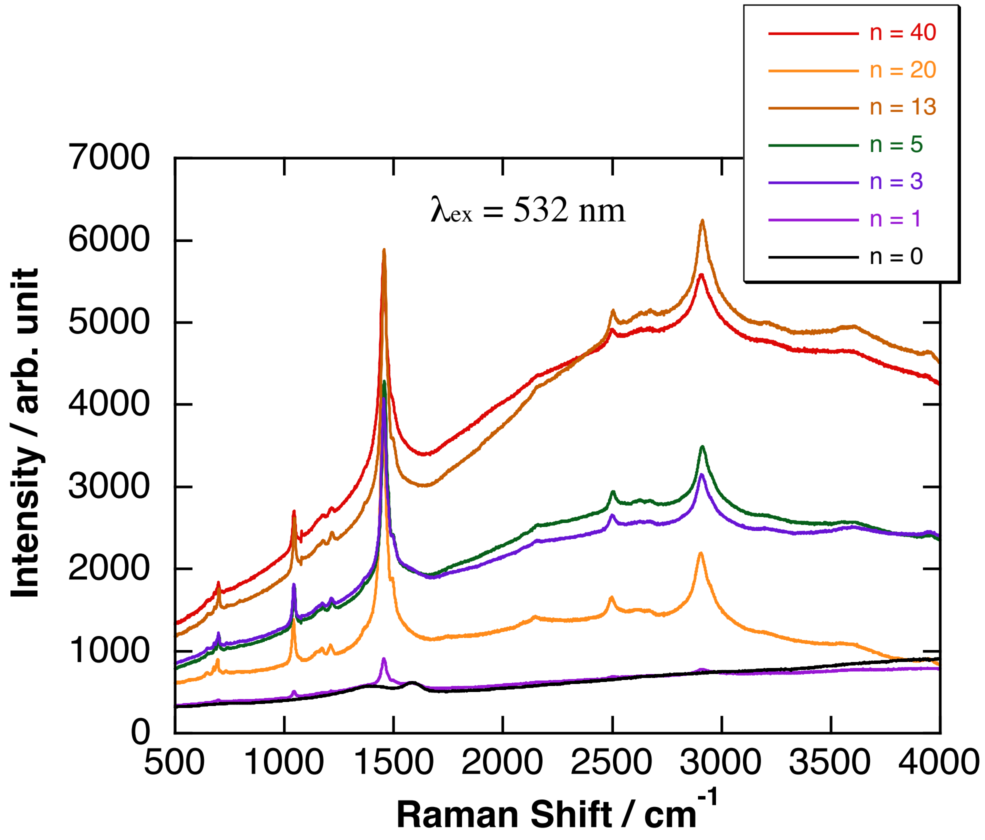

Fig. S5. Raman scattering spectra of polyBiTh(*n*)/C_{60}PF/PSS/PEI/ITO.

(a)

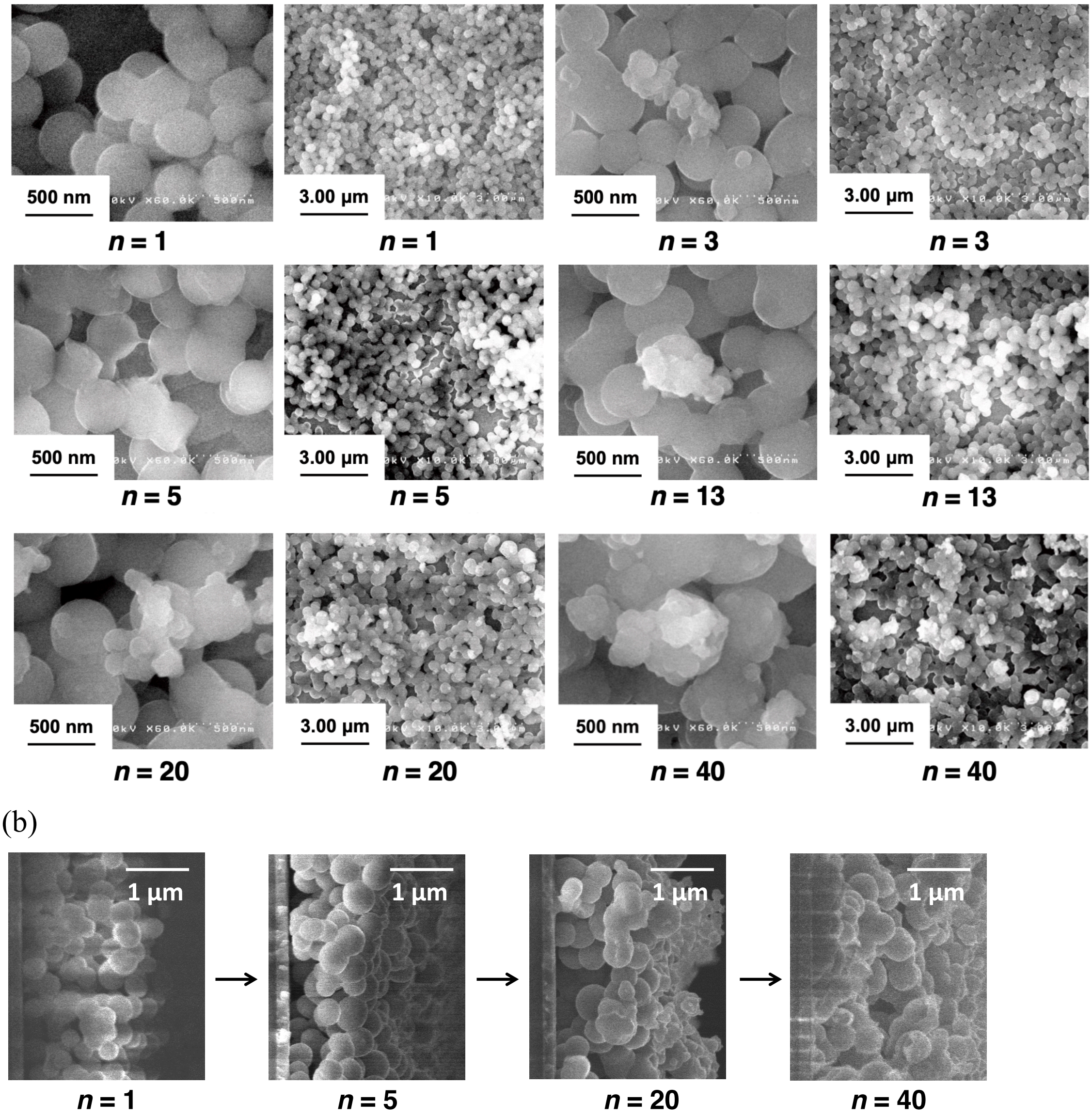

Fig. S6. (a) SEM images of polyBiTh(*n*)/C_{60}PF/PSS/PEI/ITO (*n* = 1, 3, 5, 13, 20, and 40 cycles), and (b) cross-sectional SEM images of polyBiTh(*n*)/C_{60}PF/PSS/PEI/ITO (*n* = 1, 5, 20, and 40 cycles).

(a)

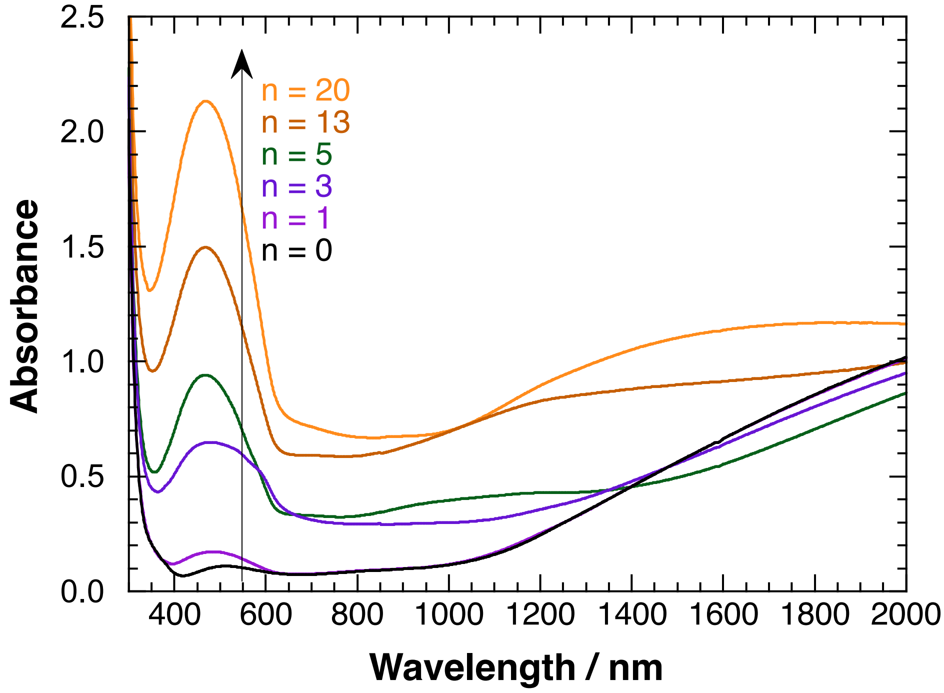

(b)

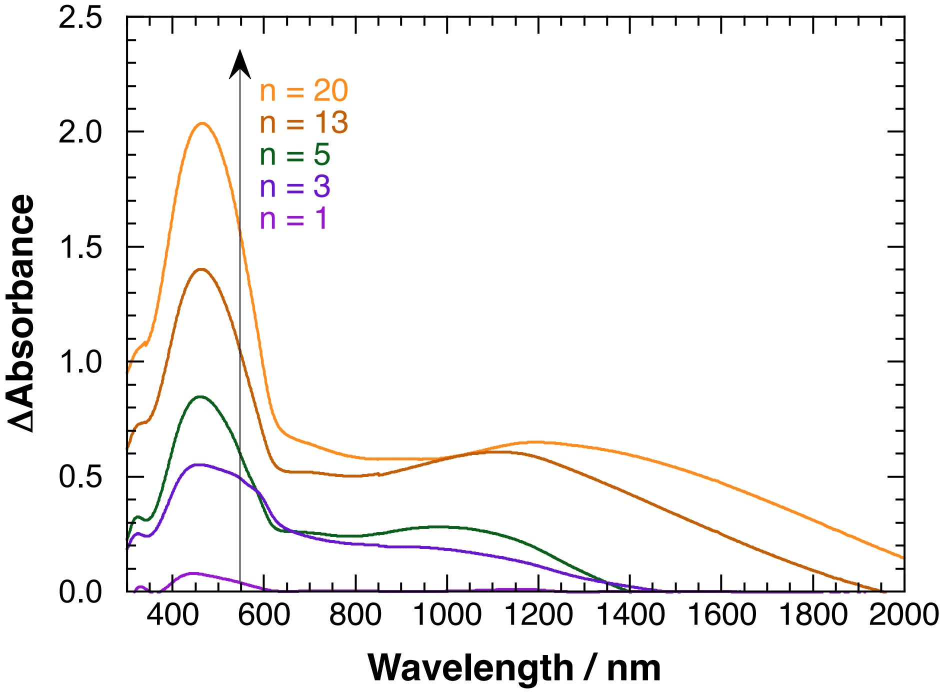

Fig. S7. (a) transmission absorption spectra of polyBiTh(*n*)/PSS/PEI/ITO, and (b) differential transmission absorption spectra of polyBiTh(*n*)/PSS/PEI/ITO after subtraction of PSS/PEI/ITO (n = 0) absorption spectra.
